# Influence of Visible Violet, Blue and Red Light on the Development of Cataract in Porcine Lenses

**DOI:** 10.3390/medicina58060721

**Published:** 2022-05-27

**Authors:** Katja Zeller, Stephan Mühleisen, Pranavi Shanmugarajah, Nicole Fehler, Robin Haag, Martin Hessling

**Affiliations:** Department of Medical Engineering and Mechatronics, Ulm University of Applied Sciences, Albert-Einstein-Allee 55, D-89081 Ulm, Germany; zellka01@thu.de (K.Z.); muehst01@thu.de (S.M.); shanpr01@thu.de (P.S.); nicole.fehler@thu.de (N.F.); robin.haag@thu.de (R.H.)

**Keywords:** cataract, porcine lens, visible light, violet light, blue light, red light, LED illuminants, transmission, dark field image

## Abstract

*Background and Objectives*: Cataract is a disease that is globally prevalent in today’s population and occurs mostly in the elderly. It is an opacity of the lens that worsens vision and can lead to blindness. One well-known risk factor of cataract is ultraviolet (UV) radiation. However, increasing exposure to modern artificial light sources like light emitting diodes (LEDs) and displays might have an impact on cataract formation due to possible high (and hidden) blue radiation. An ex-vivo study indicates that intense blue radiation causes cataract in porcine lenses. The goal of this work is the investigation whether violet or red light also lead to cataract formation in porcine lenses and to compare the impact of the different wavelengths. *Materials and Methods*: LEDs with wavelengths of 407 nm (violet), 463 nm (blue) and 635 nm (red) are used to irradiate ex–vivo porcine lenses with a dose of 6 kJ/cm^2^. Before and after irradiation the lens transmissions are measured and dark field images are taken to determine cataract formation. The same procedure is performed for unirradiated controls. *Results*: The results of the transmission measurements are in accordance with the results of the dark field images and state that 635 nm (red) is inducing no or only weak cataract. In comparison to the dark field images the transmission measurements exhibit stronger cataract formation for 407 nm than for 463 nm irradiation while the dark field images show similar cataract formation for both wavelengths. *Conclusions*: Visible light of short wavelengths cause cataract formation in porcine eyes, and it cannot be excluded that these wavelengths, which are emitted by modern LED illuminants, also pose a danger to human eyes.

## 1. Introduction

Cataract is lens opacity that worsens vision and can lead to blindness [1]. It can be treated by surgery, which is not equally accessible to the world’s population [1]. Especially in developing countries people might not have the necessary financial means or access to a surgeon who can treat cataracts [1,2]. For this reason, the causes of cataract development should be investigated in more detail. Contributing factors to the development of age-related cataract are partially understood [1]. It is known that genetic and environmental factors such as age, smoking, alcohol, diabetes, medications such as corticosteroids, malnutrition, high BMI (body mass index) and UVB radiation (ultraviolet radiation within the spectral range 280–315 nm) contribute to an increased risk of developing cataract [1,2].

The exposure to artificial light has risen strongly over the last few years [3]. The reason for this is that LED light sources are increasingly used, such as in illuminants, displays and automotive headlights [4,5]. Fluorescent tubes and energy-saving lamps have been emitting violet light for decades [6], and recently, due to the coronavirus pandemic, the use of violet LEDs in everyday life is also increasing, as light of this wavelength exhibits antimicrobial and antiviral properties [7,8,9].

Cataract is distinguishable by anatomical location, color, size and opacity. To simplify and unify cataract classification the WHO defined 3 different types of cataract: nuclear (NUC), cortical (COR) and posterior subcapsular cataract (PSC) [10,11,12]. The different types of cataract have different effects and origins. Nuclear cataract is a central opacity of the lens originating from the nucleus [10,12]. It leads to a decrease in the patient’s visual acuity [13]. In posterior subcapsular cataract, plaque-like opacifications occur in the axial posterior region of the cortical layer or centrally or paracentrally on the posterior capsule [10,12]. For this type of cataract, the patient becomes very sensitive to glare [13]. Cortical cataract usually has a wedge shape, which starts at the lens cortex and extends to the center [10,12]. Often, however, not only one form of cataract occurs in patients, but several at the same time [12]. In addition to the conventional cataract types, there are special ones due to trauma, age or developmental disorders. They are often asymmetrical and strongly separated from the rest of the tissue [14].

Since there is still no proven effective drug or other conservative treatment for cataract diseases, the only way to improve the visual acuity is surgery. During surgery, the original opaque lens is replaced by an artificial intraocular lens. The only exception to cataract surgery is cataract due to galactosemia. This is a metabolic cataract, which occurs due to the preserved galactose in the mother’s milk and can be treated non-invasively [15].

In this study, cataract formation in porcine eyes is investigated. Porcine eyes have already been applied as a model for human eyes in several experimental investigations [16,17,18]. A main reason for the selection are the phylogenetic parallels between porcine and human eye, for example, the similar thickness, shape and size of the sclera, localization of photoreceptors in the periphery of the retina or holangiotic retinal vascularization [19]. In addition, the use of ex-vivo porcine eyes involves less ethical and economic constraints, making the availability and procurement process of porcine eyes less critical compared to other species [20].

The influence of conventional light sources such as LEDs or fluorescent tubes emitting blue light has been mainly studied for its impact on the retina but has remained unexplored for the lens. Haag et al. reported that blue light with a wavelength of 460 nm has the greatest effect on cataract formation in ex-vivo porcine lenses in comparison to UVA and UVB radiation [21]. Based on these results, this work analyses and compares the impact of further visible wavelengths. The experiments are performed with LEDs of 407 nm (violet), 463 nm (blue) and 635 nm (red). These wavelengths have been selected because white LEDs exhibit strong blue peak emissions, powerful red LEDs are an essential component of all displays and illuminants with adjustable color and violet emission peaks around 405 nm are found in fluorescent and energy saving lamps and strong violet LEDs that are recently applied for disinfection purposes. Different experimental methods are applied to characterize the occurring cataract in the lenses like transmission measurements and dark field image analysis. The results of both measurement methods are compared to each other and evaluated for each wavelength.

## 2. Materials and Methods

### 2.1. Light Sources

The LED sources are selected so that an irradiance of about 70 mW/cm^2^ is achieved. For determining the irradiance on the lens surface, an optical power monitor “OPM 150” of Artifex (Emden, Germany) is placed at the position where the lens is located during irradiation. Afterwards, the OPM is replaced by the lenses, which are to be irradiated. This irradiance allows the application of a total dose of 6 kJ/cm^2^ within 24 h and a comparison with the former results of Haag et al. [21]. The following light sources are chosen. For the violet irradiation with a peak emission at 407 nm one single LED “LuxiGenTM 385–410 nm” of OSRAM (Munich, Germany) is used. For the blue irradiation with a peak emission at 463 nm four LEDs “Nichia NCSB219B-V1 SMD-LED” of Nichia (Anan, Japan) are applied and for the red spectrum two LEDs type “LZ4-00R108“ of OSRAM with a peak emission at 635 nm are chosen.

For each wavelength, a specific LED-set is built. Each set consists of a ventilator, a cooling element and one or more LEDs, which are attached to the cooling element by a heat conduction pad. The illumination of the sample area is quite homogeneous with a loss of up to 20% at the edges.

### 2.2. Experimental Setup

**Figure 1:**
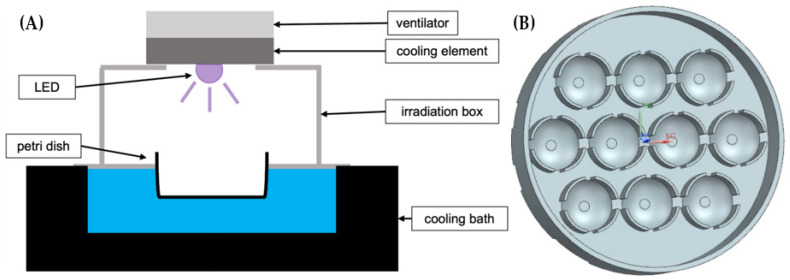
(**A**) Setup for irradiation of porcine lenses, consisting of a 3D-printed box, a cooling water bath and a LED-set with a ventilator and cooling element. (**B**) Custom-made Petri dish with ten holders for storing lenses during irradiation.

The experimental setup consists of a 3D-printed box to secure the samples from environmental light, a LED-set and a custom-made Petri dish, which stores up to ten samples and is made of black plastic to minimize reflections (see Figure 1). The bottom part of the box, which is also the holder of the Petri dishes, is placed in a cooling water bath “Thermocell Cooling & Heating Block” (model: CHB-202) of Bioer (Hangzhou, China). This allows to hold the temperature of the samples at 20 °C during the illumination. The upper cover of the box has a hole through which the LEDs illuminates the samples. A second box with a closed upper cover is applied for the unirradiated controls.

### 2.3. Experimental Procedure

The ex-vivo porcine eyes of 6 to 8 months old pigs, are obtained from a local slaughterhouse and investigated on the day of enucleation. In the first step, eyes and lenses are scanned for possible damages and already existing cataracts. Conspicuous samples are sorted out and not considered for further experiments. The lenses are carefully dissected from the eyes and stored in BSS (balanced salt solution), which is similar to aqueous humor [22]. Then transmission measurements and dark field image analyses are performed to evaluate and quantify cataract development.

These methods allow a comparison of the opacity of lenses before and after irradiation with different wavelengths. For visualizing cataract in the porcine lenses a single lens is placed in a Petri dish with a mounting disk that prevents the lens from sliding around, which is filled with BSS and a picture is taken with a dark field microscope [21]. The dark field microscope camera generates a grayscale image. The evaluation of the gray images is performed with MATLAB (MathWorks, Natick, MA, USA) according to the method of Haag et al. [21]. A histogram linearization is performed to increase the contrast of the captured original image. On this histogram linearized image, a circle is defined around the lens to be cut out. To evaluate the cataract formation, the brightness of the lens structures are determined by summing the pixel values of the cut out original image. In addition, false color images are generated from the grayscale images to improve the visualization of cataract formation.

For the transmission measurements the lenses are put in the wells of a 6x8 microtiter plate “COSTAR48”, with 500 µL of BSS in each well. The measurements are performed in a spiral, with a diameter of 5 mm from the central point of each lens with the microtiter plate reader “CLARIOstar PLUS” of BMG Labtech (Ortenberg, Germany). The transmission measurement is performed in a wavelength range of 220–1000 nm and measurements are taken in 1 nm steps. Afterwards, the samples are irradiated for 24 h by one of the LED-sets at the selected wavelength until a dose of 6 kJ/cm^2^ is reached. During this time, samples and controls are stored in custom-made Petri dishes (see Figure 1B) filled with CAS (complex antimicrobial saline). This solution is a composition of three different components: 94% BSS, 4% fetal calf serum (strong absorption at 405 nm) and 2% GVPC (Glycine, Vancomycin, Polymyxin B, Cycloheximid). CAS reduces growth of microorganisms such as fungi and bacteria and acts against the decomposition of biological samples.

During irradiation, the samples are kept at a constant temperature of 20 °C. The control group of unirradiated lenses is stored under the same conditions as the irradiated samples, but without irradiation. After the samples are irradiated for 24 h transmission measurements are repeated again and dark field images are taken.

A total of 95 lenses are irradiated. 33 lenses with a wavelength of 407 nm (violet), 34 lenses with a wavelength of 463 nm (blue), 28 lenses with a wavelength of 635 nm (red). For the control group 115 lenses are examined.

### 2.4. Transmission Analysis

The transmission spectra of the irradiated lenses are compared to each other to find out whether a lens change has occurred and how strong it is. To quantify changes in the lenses the spectra of the transmission of the lenses before and after irradiation or before and after storage for the controls are converted to an area under the curve (AUC) in the wavelength range from 400 to 750 nm. This AUC-value is applied to determine transmission changes. Therefore, first the ratio of the change of the samples (see Equation (1)) and of the controls (see Equation (2)) are calculated. A value of one means that there is no difference between the lenses before and after storage or irradiation. The closer the value is to zero, the more the lens is affected by aging or irradiation and the more the transmission spectra have changed. With Equations (1) and (2) the change of the samples, which are irradiated in comparison to the mean change of all stored control lenses taking into account the decay effect is calculated by the ratio in Equation (3).

(1) $$\mathrm{AUC}\;\mathrm{ratio}\;\mathrm{of}\;\mathrm{sample}\;\mathrm{change}=\frac{\mathrm{AUC}\;\mathrm{sample}\;\mathrm{after}\;\mathrm{irradiation}}{\mathrm{AUC}\;\mathrm{sample}\;\mathrm{before}\;\mathrm{irradiation}}$$

(2) $$\mathrm{AUC}\;\mathrm{ratio}\;\mathrm{of}\;\mathrm{control}\;\mathrm{change}=\frac{\mathrm{AUC}\;\mathrm{control}\;\mathrm{after}\;\mathrm{storage}}{\mathrm{AUC}\;\mathrm{control}\;\mathrm{before}\;\mathrm{storage}\;}$$

(3) $$R_{Trans}=\frac{\mathrm{AUC}\;\mathrm{ratio}\;\mathrm{of}\;\mathrm{the}\;\mathrm{sample}\;\mathrm{change}}{{\mathrm{mean}\;\mathrm{of}\;\mathrm{AUC}\;\mathrm{ratio}\;\mathrm{of}\;\mathrm{the}\;\mathrm{controls}\;}^{}\mathrm{change}}$$

A value of one for the overall change in Equation (3) means that there is no difference between an irradiated sample and the stored controls. If the distance to zero decreases, the more the transmission spectra has changed. This means that the effect of the irradiation to the samples has a high influence. It also considers potential decay effects of the lenses.

### 2.5. Dark Field Images

For the evaluation of the dark field images, first the average signal per pixel (*p_av_*) is determined (see Equation (4)). Therefore, the summed pixel value per area of the cut out original image is calculated with the mean radius of the lens (*r_mean_*) and the summed pixel values of the lens (*p_sum_*).

(4) $$p_{av}=\frac{p_{sum}}{\pi \;r_{mean}²}$$

The offset of the dark field image, which is determined by the mean dark value per area, is subtracted from the average signal per pixel of the irradiated and non-irradiated lenses. This true pixel value (*p_true_*) is used to determine the change in the lenses. Therefore, first the ratio of the change in pixel values of the samples after irradiation (see Equation (5)) and of controls after storage are calculated (see Equation (6)). These are used to determine the ratio *R_DFI_*, where the change in the pixel values of each sample compared to the mean change of the stored controls is calculated to account for decay effects of the lenses (see Equation (7)).

(5) $$\mathrm{Ratio}\;\mathrm{of}\;\mathrm{sample}\;\mathrm{change}=\frac{p_{true}\;\mathrm{of}\;\mathrm{sample}\;\mathrm{after}\;\mathrm{irradiation}}{p_{true}\;\mathrm{of}\;\mathrm{sample}\;\mathrm{before}\;\mathrm{irradiation}}$$

(6) $$\mathrm{Ratio}\;\mathrm{of}\;\mathrm{control}\;\mathrm{change}=\frac{p_{true}\;\mathrm{of}\;\mathrm{control}\;\mathrm{after}\;\mathrm{storage}}{p_{true}\;\mathrm{of}\;\mathrm{control}\;\mathrm{before}\;\mathrm{storage}}$$

(7) $$R_{DFI}=\frac{\mathrm{ratio}\;\mathrm{of}\;\mathrm{sample}\;\mathrm{change}}{\mathrm{mean}\;\mathrm{of}\;\mathrm{ratio}\;\mathrm{of}\;\mathrm{controls}\;\mathrm{change}}$$

The closer the ratio *R_DFI_* is to one, the smaller is the effect of cataract formation If the ratio *R_DFI_* is greater than one, irradiation has a greater impact on cataract formation than the decay effect.

### 2.6. Statistical Analysis

The statistical evaluation of the data of *R_Tran_*_s_ and *R_DFI_* is performed with IBM SPSS Statistics 25. A one-way ANOVA is performed followed by a post-hoc analysis. Depending on whether the data show variance homogeneity, the Tukey test (variance homogeneity of the data is given) or the Games-Howell test (variance homogeneity of the data is not given) is performed as post-hoc analysis.

## 3. Results

### 3.1. Transmission

**Figure 2:**
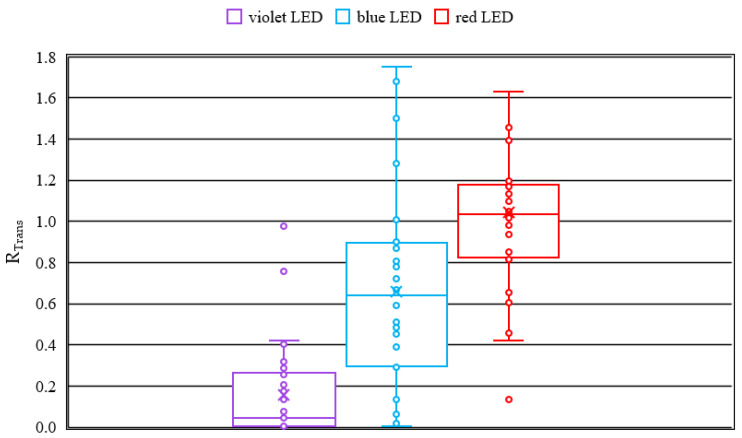
Boxplot overview of the transmission change *R_Trans_* in the visible range from 400 to 750 nm—calculated according to Formula (3).

For comparison of the transmission results of the different wavelengths, the corresponding ratios *R_Trans_*, which are calculated by Equation (3), are plotted in the boxplot (see Figure 2). No change in transmission results in a value of one. A high change in transmission causes a value closer to zero.

For irradiation with 407 nm, a median of 0.05 and a mean value with standard deviation of 0.16 ± 0.23 are found for *R_Trans_*. The upper whisker is just above 0.4 and the lower whisker is close to 0. This shows a small scatter in the transmission change. 50% of the values lie between 0.26 and 0. For irradiation with 463 nm a median of 0.64 and a mean value of 0.66 ± 0.50 are observed. The upper whisker is just below 1.8 and the lower whisker close to 0. Here a large scatter of the transmission change of the lenses is revealed. 50% of the lenses exhibit an *R_Trans_* between 0.3 and 0.9. For the irradiation with 635 nm, a median of 1.03 and a mean value of 1.04 ± 0.52 are observed. The upper whisker is just above 1.6 and the lower whisker just above 0.4. A relatively large scattering of the results is visible. Performing a one-way ANOVA reveals that *R_Trans_* is statistically lower for smaller wavelengths than for higher ones. Since variance homogeneity is not given, the Games-Howell test is performed as post-hoc test and results in *p* < 0.05 for all tests.

### 3.2. Dark Field Images

**Figure 3:**
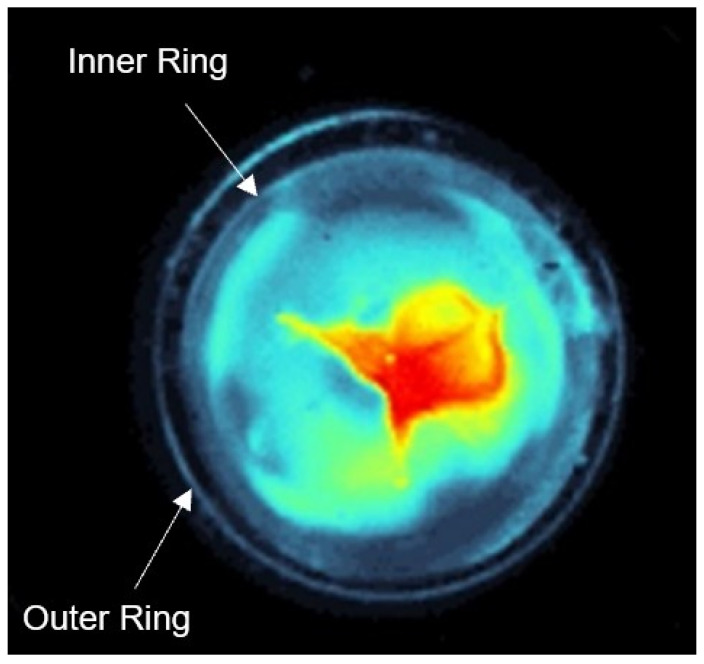
An outer and inner ring can be observed in the dark field image. The outer ring shows the edge of the mounting disk, in which the lens is stored in the medium and the inner ring represents the edge of the lens.

For each irradiation wavelength and for the controls, a representative lens was selected exemplarily to visualize the change of the lens. The corresponding dark field images were converted into false color image with the same scale, since these make the scattering of the light more visible. In all images an outer ring and an inner ring can be observed (see Figure 3). The outer ring is the edge of the mounting disk, in which the lens is stored in the medium, so that the lens is kept in the right position during the dark field imaging. The inner ring is the edge of the lens. The pixel values between the outer and the inner ring were not considered in the evaluation.

**Figure 4:**
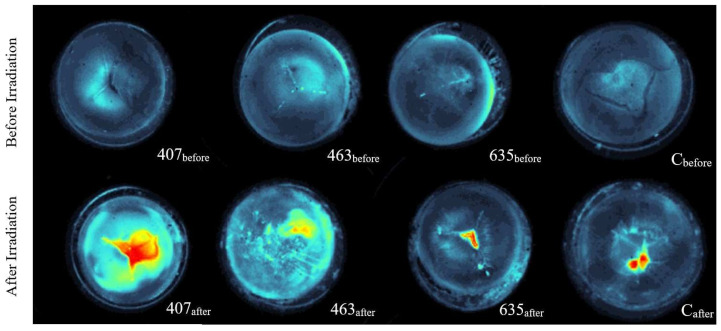
False color images converted from captured gray images. The first three lenses 407_before_, 463_before_ and 635_before_ in the upper row were taken before irradiation and in the lower row the same lenses 407_after_, 463_after_ and 635_after_ were taken after irradiation with 407 nm, 463 nm and 635 nm. The last lens C_before_ and C_after_ illustrates an unirradiated control lens. Here, a false color image was generated before and after a storage period of 24 h.

The first three images in the top row of Figure 4 illustrate the lenses before irradiation and the bottom row gives the same lenses after irradiation. The last lens exhibits the control in comparison before to after storage. The first lens (407_before_) before irradiation with 407 nm reveals a slight star cataract, but otherwise no opacity is visible. After irradiation, a strong nuclear cataract formation is observed in the lens (407_after_). The irradiation with 463 nm has induced subcapsular and cortical cataract in the lens (463_after_). In comparison, the lens before irradiation (463_before_) exhibits a more regular structure without clear opacity, but a preexisting star cataract is recognizable. Before and after irradiation with 635 nm no clear opacity of the lens can be observed. The lens (635_before_) is more transparent before irradiation. After irradiation, only a small area with increased scattering of light in the middle of the lens is induced (635_after_) and a slight nuclear cataract is visible. The last images illustrate the decay effect of the control lenses after a storage of 24 h without irradiation. It can be observed that the scattering of light after storage (C_after_) is higher than before storage (C_before_) only in the center of the lens.

**Figure 5:**
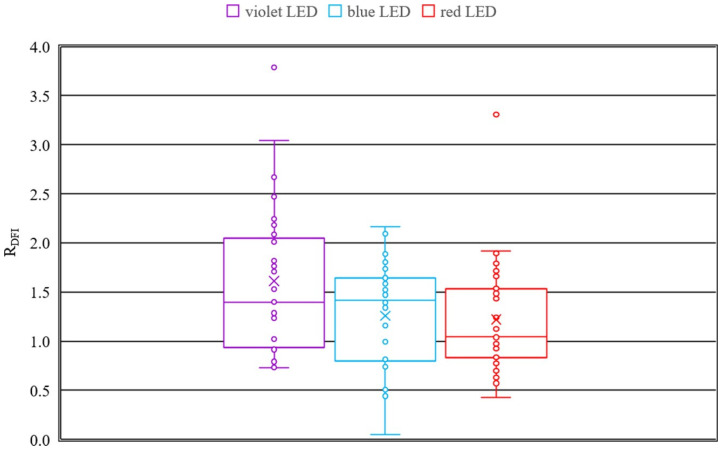
Comparison of the pixel change in the dark field images according to Equation (7) of the lenses irradiated with violet, blue and red LEDs.

To quantify the change in lenses for each wavelength, the corresponding ratios of pixel change *R_DFI_* of all lenses, calculated according to Equation (7), are presented in the boxplots in Figure 5. The ratios *R_DFI_* for the irradiation with 407 nm and 463 nm exhibit similar median values with 1.40 and 1.41 but the mean value with standard deviation at 407 nm of 1.61 ± 0.83 is higher than the mean value at 463 nm of 1.26 ± 1.52. 50% of the ratios *R_DFI_* at 407 nm are between 0.93 and 2.05 and those at 463 nm are between 0.80 and 1.64. The maximum change of *R_DFI_* = 3.04 after 407 nm irradiation is higher compared to the maximum change of *R_DFI_* = 2.17 after 463 nm irradiation. The mean and median values for the results of the irradiation with the red LED are 1.22 ± 0.55 and 1.05, respectively. 50% of the quotient values are between 0.83 and 1.53. The lens with the maximum change after irradiation with 635 nm is indicated with the ratio *R_DFI_* of 1.92. The scattering in the pixel change is higher for 407 nm and 463 nm in comparison to 635 nm. Outliers can be observed within the results of all three wavelengths. Performing a one-way ANOVA reveals that there are no statistical differences in *R_DFI_* for different wavelengths. Since variance homogeneity is given the Tukey test is performed as post-hoc test and results in *p* < 0.05 for all tests. Nevertheless, the mean value of *R_DFI_* decreases with increasing wavelength.

## 4. Discussion

The boxplots for the transmission change due to irradiation (see Figure 2) reveal, that irradiation with a wavelength of 407 nm (violet) exhibits the strongest cataract formation. This is proven by the mean, which is nearly zero. The results for the wavelength of 463 nm (blue) reveal a wide scattering of the ratios *R_Trans_*. The induced cataract ranges from nearly no cataract to very strong opacity in the lens and are in agreement with the previously reported results for blue light [21]. At 635 nm (red) the results show no up to a slight cataract formation, which is represented by a mean near to one.

By the results of the dark field image analysis the conclusion can be drawn, that irradiation with all three wavelengths is inducing cataract since the ratio *R_DFI_* is higher than one. Considering the median of the boxplots, which is more robust against outliers than the mean, it can be stated, that irradiation at 407 nm and 463 nm induce similar degreed of cataract and in contrast the irradiation at 635 nm induces no to weak opacity in the lenses.

The results of the transmission measurements are in accordance with the results of the dark field images, except of the violet irradiation, since for the irradiation with 407 nm the transmission measurement shows stronger cataract formation in comparison to the dark field images. Possible reasons are a very strong scattering and shielding of the light, which result in lower pixel values for the dark field images.

These investigations have been performed with ex-vivo porcine eyes and high irradiation doses of 6 kJ/cm^2^. It is a reasonable question whether these results can be transferred to in-vivo human eyes and whether human eyes are exposed to such doses under realistic circumstances. Unfortunately, we cannot answer these questions for human eyes, but Wang et al. investigated the effect of blue light (460 nm) on in-vivo rat eyes [23]. For 4 to 12 weeks they kept rats under a blue illumination of 3000 lux for 12 h a day and observed cataract formation. Even the shortest period of 4 weeks of 3000 lux @ 460 nm is equivalent to a total dose of blue light of about 9 kJ/cm^2^ and in the same order of magnitude as the here applied of 6 kJ/cm^2^. (The conversion from lux or lm/m^2^ to W/m^2^ and J/cm^2^ was performed with the help of the standard conversion formula for monochromatic light sources φ_v_ = 683 lm/W V(λ) φ_e_, with the luminous flux φ_v_ in lm, the radiant flux φ_e_ in W and the luminous efficiency V(460 nm) = 0.06).

Therefore, at least ex-vivo porcine and in-vivo rat eyes don’t appear to be totally different regarding cataract formation by blue light and it seems to be reasonable that in-vivo human eyes or lenses might exhibit a similar risk of cataract formation by blue and probably also by violet (LED) light.

## 5. Conclusions

Short wavelengths visible (violet and blue) light causes cataract formation in ex-vivo porcine eyes. Though there is still no prove, this might also happen to human eyes. This is important as modern artificial light sources like LEDs or energy-saving lamps with their strong blue and violet emissions are more and more replacing filament bulbs that exhibit only weak blue emissions. Therefore, though these modern illuminants do not emit ultraviolet radiation, which is known for its cataract generation, they still might lead to additional cataracts and cataract surgery.

## Data Availability

The data is available upon reasonable request.
